# The β-Lactamase Inhibitor Boronic Acid Derivative SM23 as a New Anti-*Pseudomonas aeruginosa* Biofilm

**DOI:** 10.3389/fmicb.2020.00035

**Published:** 2020-02-07

**Authors:** Samuele Peppoloni, Eva Pericolini, Bruna Colombari, Diego Pinetti, Claudio Cermelli, Francesco Fini, Fabio Prati, Emilia Caselli, Elisabetta Blasi

**Affiliations:** ^1^Department of Surgical, Medical, Dental and Morphological Sciences With Interest in Transplant, Oncological and Regenerative Medicine, University of Modena and Reggio Emilia, Modena, Italy; ^2^Centro Interdipartimentale “Grandi Strumenti” (CIGS), University of Modena and Reggio Emilia, Modena, Italy; ^3^Department of Life Sciences, University of Modena and Reggio Emilia, Modena, Italy

**Keywords:** boronic acids, *Pseudomonas aeruginosa* biofilm, quorum sensing, virulence factors, inhibitors

## Abstract

*Pseudomonas aeruginosa* is a Gram-negative nosocomial pathogen, often causative agent of severe device-related infections, given its great capacity to form biofilm. *P. aeruginosa* finely regulates the expression of numerous virulence factors, including biofilm production, by Quorum Sensing (QS), a cell-to-cell communication mechanism used by many bacteria. Selective inhibition of QS-controlled pathogenicity without affecting bacterial growth may represent a novel promising strategy to overcome the well-known and widespread drug resistance of *P. aeruginosa*. In this study, we investigated the effects of SM23, a boronic acid derivate specifically designed as β-lactamase inhibitor, on biofilm formation and virulence factors production by *P. aeruginosa*. Our results indicated that SM23: (1) inhibited biofilm development and production of several virulence factors, such as pyoverdine, elastase, and pyocyanin, without affecting bacterial growth; (2) decreased the levels of 3-oxo-C_12_-HSL and C_4_-HSL, two QS-related autoinducer molecules, in line with a dampened *lasR*/*lasI* system; (3) failed to bind to bacterial cells that had been preincubated with *P. aeruginosa-*conditioned medium; and (4) reduced both biofilm formation and pyoverdine production by *P. aeruginosa* onto endotracheal tubes, as assessed by a new *in vitro* model closely mimicking clinical settings. Taken together, our results indicate that, besides inhibiting β-lactamase, SM23 can also act as powerful inhibitor of *P. aeruginosa* biofilm, suggesting that it may have a potential application in the prevention and treatment of biofilm-associated *P. aeruginosa* infections.

## Introduction

*Pseudomonas aeruginosa* is a Gram-negative opportunistic pathogen, causing nosocomial infections in more than 2 million patients every year (Cross et al., 1983; Rossolini and Mantengoli, 2005). Such infections are particularly frequent in immunocompromised patients, such as those with cancer, AIDS, burn wounds, and indwelling devices (Vandeputte et al., 2010; Sarabhai et al., 2015). Notably, *P. aeruginosa* infections are associated with unfavorable outcome in individuals with cystic fibrosis (CF), the most common life-limiting genetic disorder in the U.S. (Lyczak et al., 2002). These patients, indeed, show abnormalities in their lung tissue that promote bacterial colonization, which in turn causes long-lasting inflammation, lung injury, and eventually respiratory failure.

*P. aeruginosa* has a uniquely large genome, whose genes encode for several virulence factors (including LasA protease, LasB elastase, pyoverdine, pyocyanin, alginate, and exotoxin A) and regulatory mechanisms, allowing microbial adaptation to many hostile environments (Stover et al., 2000). As a consequence, *P. aeruginosa* is ubiquitous in nature and almost impossible to eliminate from hospitals. As many other microorganisms, besides living in a planktonic form, *P. aeruginosa* is able to form biofilm on medical implants or damaged tissues (Xu et al., 2013). Particularly, more than 70% of hospital-acquired infections are associated with biofilm on catheters, ventilator tubes, implants, and medical prosthetic devices (Brooun et al., 2000). Unlike planktonic cell counterpart, bacterial communities structured as biofilms exhibit an altered phenotype, with respect to growth rate, expression of virulence factors, and cell-to-cell communication system (Qu et al., 2016). Thus, when enclosed in a self-produced extracellular polymeric matrix, *P. aeruginosa* is protected from patient’s immune system and becomes up to 1,000 times more antibiotic resistant than the planktonic counterpart (Mah and O'Toole, 2001).

The expression of many virulence factors in *P. aeruginosa* is controlled by the Quorum Sensing (QS) system (Venturi, 2006), an intercellular communication mechanism that coordinates bacterial behavior and gene expression by means of signaling molecules in a cell density-dependent manner (Schuster and Greenberg, 2006). The transcriptional regulation of numerous virulence genes in *Pseudomonas* is under the control of two N-acyl homoserine lactone (AHL)-dependent QS systems, named *lasI*/*lasR* and *rhlI*/*rhlR*. In the *lasI*/*lasR* system, *lasI* encodes the synthesis of N-(3-oxo-dodecanoyl)-homoserine lactone (3-oxo-C_12_-HSL), which binds and activates the cognate response regulator LasR, leading to regulation of gene expression. Differently, in the *rhlI/rhlR* system, *rhlI* synthesizes the N-(butanoyl)-homoserine lactone (C_4_-HSL), which, in turn, by interacting with the cognate RhlR, influences the transcription of target genes. Importantly, the two QS systems are hierarchically organized, with the *lasI/lasR* system in turn regulating the transcription of *rhlI/rhlR*.

These QS systems are among the most studied in bacteria and their regulons are essential for the pathogenicity of *P. aeruginosa* (Schuster and Greenberg, 2006; Girard and Bloemberg, 2008; Zou and Nair, 2009). There is also a third self-inducing signal, referred to as *Pseudomonas* quinolone signal, that plays an integral role in the QS system (Pesci et al., 1999). Such complicated communication systems coordinate/regulate several virulence traits in *P. aeruginosa*, including motility, toxin production, and biofilm formation (Fuqua and Greenberg, 1998; de Kievit and Iglewski, 2000; Dong et al., 2001; Donabedian, 2003; Jakobsen et al., 2013). Recently, a fourth intercellular communication signal has been discovered, as being capable of integrating environmental stress cues with the QS network. Named as IQS, it belongs to a new class of QS signal molecules and has structurally been established to be 2-(2-hydroxyphenyl)-thiazole-4-carbaldehyde (Lee and Zhang, 2015).

The difficulty in treating biofilm-associated *P. aeruginosa* infections has encouraged the extensive use of antibiotics, in turn facilitating the development of multiple drug-resistant strains. Among several mechanisms of antibiotic resistance, the production of *β*-lactamases, enzymes able to hydrolyze *β*-lactams, is consistently the most concerning one (Bush and Bradford, 2016). In *Pseudomonas*, class C *β*-lactamases confer high level of resistance to penicillins, cephalosporins, and monobactams (Berrazeg et al., 2015). Besides modifying the structure of *β*-lactams, leading to generation of several new penicillins and cephalosporins, another relevant strategy to overcome resistance to these drugs is the co-administration of the β-lactam antibiotic together with a β-lactamase inhibitor (Bush and Bradford, 2019). Accordingly, in the last decade, new classes of *β*-lactamase inhibitors have entered the market giving the opportunity to restore the activity of several *β*-lactams (Bush and Bradford, 2019). One of the most promising class of new *β*-lactamase inhibitors are the boronic acids transition state analog inhibitors (BATSIs), which are known to restore the *β*-lactam activity both *in vitro* and *in vivo* (Drawz et al., 2011; Eidam et al., 2012; Barnes et al., 2018). Recently, a new combination of a boronic acid and the *β*-lactam meropenem (Vabomere®) has entered the market for treatment of infections caused by carbapenem-resistant *Enterobacteriaceae* and multidrug-resistant (MDR) *Pseudomonas* (Griffith et al., 2019). In order to obtain highly active BATSIs, we have undertaken a biomimetic approach: the boronic acid structure was decorated with chemical groups arranged in a specific stereochemistry resembling that of the natural substrate *β*-lactam. Among different synthesized BATSIs, one of the best inhibitors ever tested was the SM23 (Morandi et al., 2003). This compound has an R1 acylamino side chain of the *β*-lactam cephalothin on the boron carbon α (red colored in Supplementary Material), and a meta-carboxybenzyl side chain, that mimics the 6-carboxydihydrothiazine ring of the antibiotic (blue colored in Supplementary Material). Few commercially available phenylboronic acids have also been investigated in *Vibrio harveyi*, where they act as QS inhibitors with IC50 values in the low to sub-micromolar range (Ni et al., 2009).

In this study, we investigated the effects of the β-lactamase inhibitor boronic acid derivative SM23 on biofilm formation and production of QS-dependent virulence factors as well as autoinducer molecules by *P. aeruginosa* during biofilm formation. Overall, our results provide the first evidence on the efficacy of SM23 as a remarkable anti-biofilm agent and QS inhibitor, envisaging its use in the prevention and treatment of biofilm-associated *P. aeruginosa* infections.

## Materials and Methods

### SM23

The boronic acid SM23 was synthesized as previously described (Morandi et al., 2003). The compound was dissolved in DMSO and the different working concentrations were prepared by diluting each stock solution in PBS. In all experiments, the final DMSO concentration was less than 0.1%.

### Microbial Strain

We used the bioluminescent *Pseudomonas aeruginosa* strain (P1242), previously engineered to express both the luciferase gene and substrate under the control of a constitutive P1 integron promoter, in order to constitutively produce a detectable bioluminescent signal (Choi and Schweizer, 2006).

Bacterial cells from −80°C glycerol stocks were initially seeded onto Tryptic Soy Agar (TSA) plates and incubated overnight at 37°C; isolated colonies were then collected, added to 10 ml of Tryptic Soil Broth (TSB), and allowed to grow overnight at 37°C with gentle shaking. Bacterial concentrations were assessed by the McFarland standard curve and diluted to the required experimental concentration.

### Biofilm Formation and Quantification

To investigate the effect of SM23 on *Pseudomonas* biofilm formation, a bacterial cell suspension (10^8^ cells/ml in TSB plus 2% sucrose) was seeded (100 μl/well) in a 96-well microtiter plate (Sarstedt, Nümbrecht, Germany), treated or not with 100 μl of scalar doses of SM23 (range concentrations from 0.390 to 25.0 μM) and incubated for 24 h at 37°C to allow biofilm formation. After incubation, each well was gently washed to remove planktonic cells, then crystal violet (CV) staining was performed to quantify biofilm formation as previously described (Stepanovic et al., 2000). The absorbance at 570 nm was spectrophotometrically measured by the SunRise Microplate Reader (Tecan Group Ltd., Männedorf Switzerland). The results were expressed as optical density (OD_570_) mean ± SEM of the biofilm biomass. In order to evaluate the metabolic activity of *Pseudomonas* biofilm, 100 μl of bacterial culture in TSB + 2% sucrose (5 × 10^4^ cells/ml) were seeded (100 μl/well) in a 96-black well microtiter plate, treated or not with scalar doses of SM23 at concentrations ranging from 0.390 to 25.0 μM (100 μl/well) and then incubated for 24 h at 37°C to allow biofilm formation. After incubation, each well was gently washed twice with PBS (EuroClone, Whethereby, UK) to remove planktonic cells and then bioluminescence signal was measured by Viktor Luminescence reader (Perkin Elmer). The results were expressed as Relative Luminescence Units (RLU) mean ± SEM of metabolically active biofilm.

### Confocal Microscopy Analysis of *P. aeruginosa* Biofilm

The fluorescence property of *Pseudomonas* spp. (Meyer, 2000; Cornelis and Matthijs, 2002) was exploited to perform confocal microscopy imaging. Briefly, bacterial suspensions (1 × 10^5^ cells/ml in TSB plus 2% sucrose) were seeded on coverslips (1,000 μl/well) inserted into 24-microplate (Sarstedt, Nümbrecht Germany) and treated or not with SM23 at 0.780 and 3.125 μM. The plates were then incubated for 24 h at 37°C to allow biofilm formation. After incubation, the coverslips were washed twice with PBS, fixed with 4% paraformaldehyde (PFA) (Sigma-Aldrich, Darmstadt) for 30 min at 4°C, washed again and then analyzed by confocal microscope Leica TCS SP8 (Wetzlar, Germany) at excitation/emission wavelength 492/517 nm.

### Evaluation of Live or Dead Bacterial Cells in Biofilm

In order to evaluate the live or dead cells in *Pseudomonas* biofilm, the bacterial cells (1 × 10^8^ cells/ml) were seeded in 96-well black-plates and treated or not with 0.780 and 3.125 μM of SM23 for 24 h at 37°C to allow biofilm formation. After incubation, the samples were stained with the “live/dead cells stain kit” (Thermo Fisher Scientific, Waltham, Massachusetts, USA), using 5(6)-carboxyfluorescein diacetate (CFDA) to label alive cells (30 min at 37°C plus 5% CO_2_) and propidium iodide (PI) to stain the dead cells (15 min at 37°C plus 5% CO_2_). The staining procedure was conducted according to the manufacturer’s instructions. After incubation, the samples were washed twice with PBS and the fluorescence emission (CFDA excitation/emission: 485/528; PI excitation/emission: 528/645) was analyzed using a multi-well fluorescence plate reader (Synergy HTX, BIOTEK, Winooski, Vermont, USA). The results were expressed as Relative Fluorescence Units (RFU) mean ± SEM of alive/dead biofilm cells.

### Elastase Activity

Elastase activity was measured in cell-free supernatants from SM23 *Pseudomonas* cells, treated or not with the compound (concentration range from 0.780 to 3.125 μM), during biofilm formation. After 6, 12, and 24 h of culture, the elastase activity was measured as described by (Ohman et al., 1980) using Elastin-Congo Red (ECR) (Sigma, St. Louis, USA) as a substrate. Briefly, 100 μl of untreated or SM23-treated supernatants were mixed with 900 μl of ECR buffer (100 mM Tris, 1 mM CaCl2, pH 7.5) containing 20 mg of ECR and then incubated for 3 h at 37°C. The reaction was terminated by adding 1 ml of 0.7 M sodium phosphate buffer (pH 6.0) and the tubes were placed in cold water bath. The insoluble ECR was removed by centrifugation at 10,000 rpm for 10 min and then the absorbance was measured at 495 nm by a SunRise Microplate Reader. The elastase activity was expressed as the optical density (OD_495_) mean ± SEM.

### High-Performance Liquid Chromatography-Mass Spectrometric Analysis

Pyoverdine, pyocyanin, 3-oxo-C_12_-HSL and C_4_-HSL molecules were assessed in culture supernatants of *P. aeruginosa* during biofilm formation after 6, 12, and 24 h of SM23 treatment. All supernatants for the high-performance liquid chromatography-mass spectrometric (HPLC-MS) analysis were filtered on Amicon Ultra-0.5 10 K centrifugal filter devices and 1:5 diluted with 5% methanol - 0.2% formic acid in MilliQ water. The HPLC-MS instrument used was an UltiMate 3,000 system, consisting of an online degasser, a Binary Pump HPG 3400RS, a Well Plate Autosampler WPS 3000RS, and a Thermostatted Column Compartment TCC 3000RS coupled to a Q-Exactive hybrid quadrupole – orbitrap mass analyzer *via* a HESI-II heated electrospray ion source (Thermo Scientific). Chromatographic separation of a 5 μl sample injection was performed on a Poroshell 120 SB-C18 100 × 2.1 mm ID, 2.7 μm ps column (Agilent) at 30°C and a 0.4 ml/min flow rate. A linear gradient elution scheme was used with mobile phase components being 0.1% formic acid in water (A) and methanol (B). The gradient started at 2% B which was maintained for 0.5 min, then raised up to 30% B in 30 min, and up again to 98% B in 24.5 min. The column was then kept at 98% B for 17.9 min, then starting conditions were restored in 0.1 min and maintained for 19 min pending a successive injection. Electrospray ionization was operated in positive ion mode, using nitrogen as sheath gas (50 arbitrary units), auxiliary gas (290°C, 40 arbitrary units), and sweep gas (3 arbitrary units). The sprayer voltage was kept at 3.8 kV and the transfer capillary temperature was set at 320°C. The Q-Exactive was operated in Full MS/dd-MS2 mode. The Full MS scan range was set from *m*/*z* 170 to 1,000 at 70,000 FWHM resolution (*m*/*z* 200). The automatic gain control (AGC) target was set at 1.0 × 10^6^ with a maximum injection time (IT) of 200 ms. Data-dependent MS2 (dd-MS2) acquisitions at 17,500 FWHM resolution (*m*/*z* 200) were triggered for the Top 3 precursor ions following each Full MS scan. The intensity threshold for precursor ion selection was set to 1.0 × 10^5^, then dynamic exclusion was active for 20.0 s. AGC target and maximum IT for the MS2 experiments were set to 2.0 × 10^5^ and 50 ms. Each precursor ion was fragmented using stepped normalized collision energy (NCE) values at 28, 50, and 75.

### *lasI*/*lasR* Gene Transcription Analysis

A qRT-PCR was used to investigate SM23-induced changes in transcription levels of selected QS genes. Briefly, *Pseudomonas* cells (1 × 10^7^ cells/ml) were seeded in 24-well flat-bottom plates (Becton Dickinson Labware Europe, Meylan Cedex, France) and untreated or treated with SM23 (1.56 μM) for 24 h at 37°C to allow biofilm formation. After incubation, each well was washed twice with PBS to remove the no-adherent bacterial cells; then, biofilm was recovered by scraping and subsequent centrifugation at +4°C; the pellet was dried and stocked at −80°C until qRT-PCR analysis. Total RNA was extracted using the HiPurA Bacterial RNA Purification (Himedia) kit and treated with DNase I on a column to remove the DNA. RNA was quantified by MaestroNano spectrophotometer readings for microvolumes. Two micrograms of total RNA and random primers were used for cDNA synthesis using EasyScript cDNA Synthesis (Abm) kit. Expression of *lasI*, *lasR*, and *16S* rRNA genes was performed in triplicate by real-time PCR using 20 ng of cDNA, 0.5 μM of forward and reverse primers and BrightGreen 2x qPCR MasterMix (Abm). The primers list is shown in Supplementary Material. The amplification conditions were the following: 1 cycle for 10 min at 95°C; 40 cycles for 15 s at 95°C, 1 min at 60°C; melting curve for 30 s at 95°C, 30 s at 65°C, and 30 s at 95°C. The expression levels of the target genes in treated samples were normalized to the expression of the reference 16S rRNA gene and then compared to the control samples. The relative expression levels were calculated using the 2^−ΔΔCt^ method.

### SM23 Interaction With Bacterial Cells

**Figure 1:**
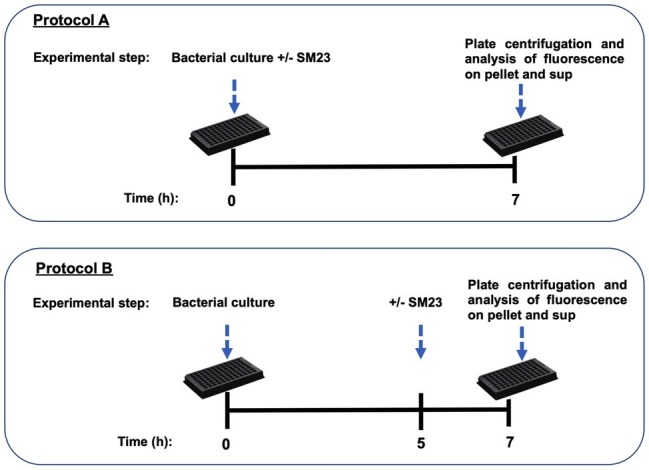
Time-lines of the experimental protocols used (protocol A and protocol B), as detailed in section “Materials and Methods.”

Two experimental protocols, A and B, were used. Bacterial cell cultures (1 × 10^8^ cells/ml; 200 μl/well) were seeded in a 96-black microtiter plate and incubated for 7 h at 37°C and 5% CO_2_, in the presence of SM23 (12.5 μM), as illustrated by the time-line of protocol A (Figure 1). In parallel experiments, microbial cells were seeded in a 96-black well microtiter plate and incubated for 5 h at 37°C and 5% CO_2_; then, SM23 was added to the cells and the plates were further incubated for 2 h, according to the time-line of protocol B (Figure 1).

In selected experiments, fresh bacterial cells were suspended at the concentration of 1 × 10^8^ cells/ml and pre-treated for 30 min with cell-free supernatants (*P. aeruginosa-*conditioned medium, recovered from an overnight culture of *Pseudomonas* at the starting concentration of 1 × 10^8^ cells/ml). To exclude residual viable bacteria in such *P. aeruginosa-*conditioned medium, 50 μl of the supernatants was seeded onto TSA plates and incubated for 48 h at 37°C under aerobic conditions; no bacterial CFUs on TSA plates were ever observed. Bacterial cells were then incubated for 7 h with the SM23 (12.5 μM), as described in protocol A. Control wells (namely, medium alone, medium plus SM23, and untreated microbial culture) were also included in the assay to evaluate the SM23 fluorescent signal. In all the experimental protocols, at the end of the incubation period (7 h), the plates were centrifuged for 10 min at 5,000 rpm. The recovered supernatants were then collected, transferred in new wells, and the fluorescent signal in both pellets and supernatants was measured by a multi-well fluorescence plate reader (Synergy HTX, BIOTEK, Winooski, Vermont, USA). The residual fluorescence of SM23 in the cell pellets and cell-free supernatants was defined as the fluorescence values obtained by subtracting, respectively, the fluorescence of the pellet or supernatant, observed in the untreated control groups, from the fluorescence values of the corresponding treated samples. The resulting values were expressed as % of residual fluorescence of SM23 detected in the pellet versus the supernatant.

### Biofilm Formation on Endotracheal Tubes and Quantification of Pyoverdine Release

Two-hundred microliter of overnight culture of *Pseudomonas* (5 × 10^4^/ml) in TSB with 2% sucrose were seeded in 96-well black-plates, containing 1 endotracheal tube (ETT) piece/well; the plates were then incubated for 90 min at 37°C (adhesion period). After incubation, the ETT pieces were washed twice with PBS, transferred into new wells, and incubated for further 24 h at 37°C in fresh TSB medium in the presence of SM23 (1.560 and 3.150 μM) to allow biofilm formation. At the end of incubation, the ETT pieces were removed, washed twice with PBS, and the bioluminescence signal was measured by Viktor Luminescence reader (Perkin Elmer). The values were expressed as Relative Luminescence Units (RLU). In parallel, after removing ETT pieces, the supernatants were recovered and centrifuged twice (10,000 rpm for 15 min) to remove the remaining bacteria. Pyoverdine release was quantified in 100 μl of culture supernatants and fluorescence emission was measured with a multi-well fluorescence plate reader (excitation/emission: 360/460), according to a standard protocol (Das et al., 2016). The values were expressed as RFU.

### Statistical Analysis

Quantitative variables were tested for normal distribution. Statistical differences between groups were analyzed according to one-way ANOVA or Kruskal-Wallis followed by Dunnett’s multiple comparisons tests by using GraphPad prism 8. A value of *p* ≤ 0.05 was considered significant.

## Results

### Inhibitory Effects of SM23 on *Pseudomonas aeruginosa* Biofilm Formation

**Figure 2:**
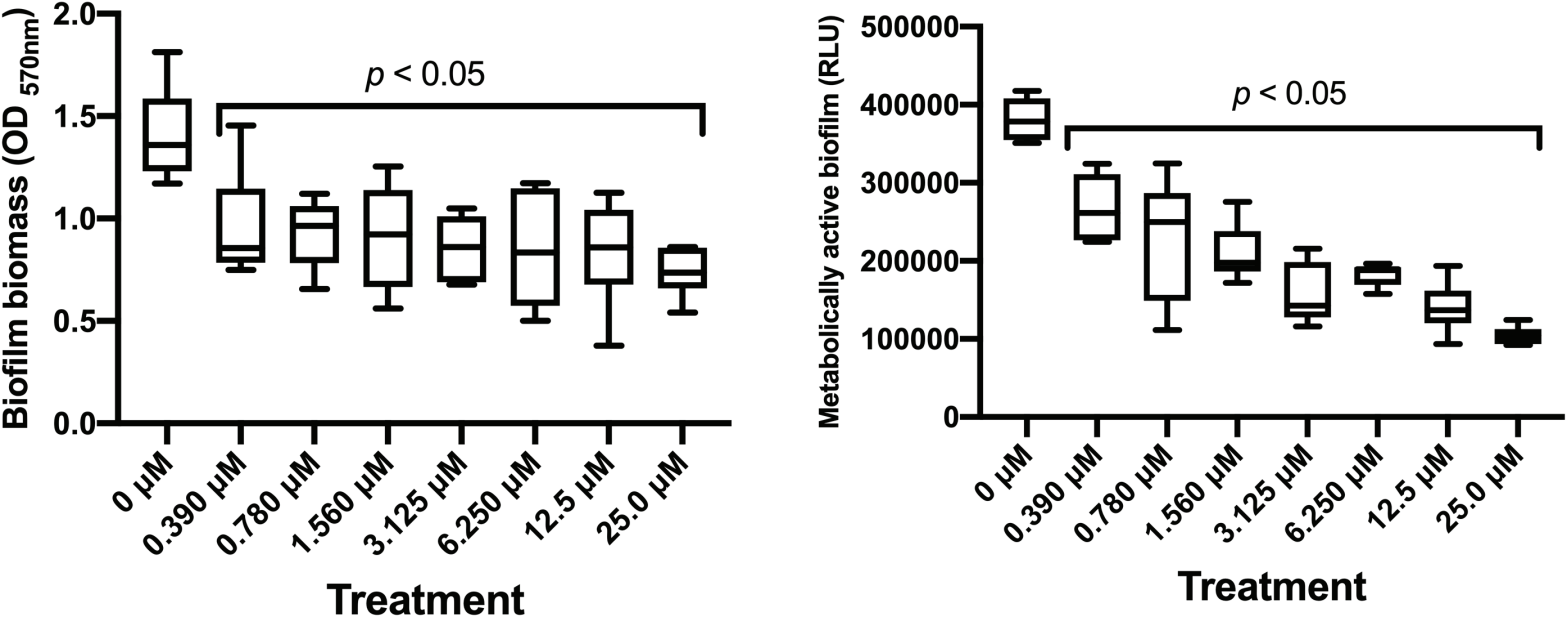
Dose-dependent effect of SM23 on *P. aeruginosa* biofilm formation. Both box-plot graphs show the mean ± SEM of microbial biofilm produced after 24 h of incubation in medium or in the presence of scalar doses of SM23. Left panel: mean ± SEM of the OD_570nm_ (biofilm biomass) of triplicate samples from two different experiments. Right panel: mean ± SEM of the Relative Luminescence Units (RLU) of triplicate samples from two different experiments. *p* < 0.05; SM23-treated vs. untreated according to one-way ANOVA followed by Dunnett’s multiple comparisons test.

Dose-dependent experiments were performed to assess the ability of SM23 to interfere with the biofilm formation by *P. aeruginosa*. Microbial cells were grown in a 96-well microtiter plate in medium only or in the presence of scalar doses of SM23 and then incubated for 24 h to allow biofilm formation. After incubation, each well was gently washed to remove planktonic cells and then CV staining was performed to quantify the biofilm biomass. The results shown in Figure 2 (left panel) indicated that the SM23, at the dose of 0.390 μM, was already able to significantly inhibit biofilm formation (about 40% of biofilm biomass), achieving its greatest effect in the dose range of 0.780–6.250 μM (about 50% of biomass reduction observed). Next, we analyzed the inhibitory effect of SM23 on biofilm, exploiting the bioluminescent properties of *P. aeruginosa* strain P1242, a model that allows to evaluate the effects of a given compound directly on the metabolically active biofilm (Pericolini et al., 2018). Thus, bacterial biofilm was allowed to form in 96-black well microtiter plates without or with SM23 at different concentrations. After 24 h of incubation, each well was gently washed to remove planktonic cells and, then, the RLU emitted by the metabolically active bacteria embedded in biofilm were measured. Results in Figure 2 (right panel) indicated that SM23 significantly reduced biofilm formation in a dose-dependent manner (from 0.390 to 25 μM concentration). In particular, 1.560 and 3.125 μM were the lowest SM23 doses still capable of reducing biofilm by 50–60%. Thus, from now on, we mainly used these two concentrations.

In parallel experiments, the influence of SM23 was evaluated on the growth of *P. aeruginosa*, cultured in planktonic form for 7 and 24 h. We found that SM23 failed to induce any change on bacterial growth capacity and viability as well, up to the concentration of 25.0 μM; also, the bacterial doubling times were found to be comparable, in SM23-treated and untreated controls (data not shown).

### Morphological Observation of SM23-Treated Biofilm

**Figure 3:**
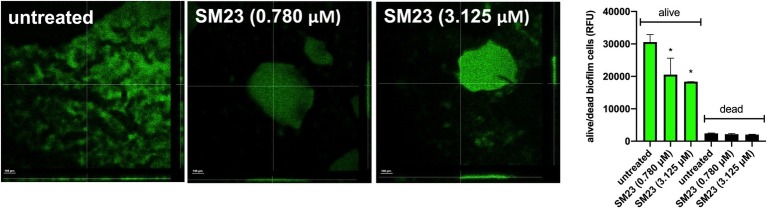
Confocal analysis of SM23-treated biofilm and evaluation of dead/alive cells. **Left panel:** confocal microscope images of a 24 h-old biofilm, untreated or treated with SM23 (0.780 and 3.125 μM). The large images illustrate three representative horizontal sections (*X* and *Y* axes) of biofilms, while the small images show biofilm sections observed along the *Z* axis. The micrographs are representative of two independent experiments. Scale bar: 100 μm. Magnification: 20x. **Right panel:** mean ± SEM of the Relative Fluorescence Units (RFU) of alive (green columns) and dead (black column) cells in a 24 h-old *Pseudomonas* biofilm treated with SM23 (0.780 and 3.125 μM). ^*^p < 0.05; SM23-treated vs. untreated according to one-way ANOVA followed by Dunnett’s multiple comparisons test.

We next performed confocal analysis in a 24 h-old biofilm, produced in medium alone or in the presence of SM23, at 0.780 and 3.125 μM. As expected, both doses strongly affected biofilm development and its morphology. Indeed, as shown in Figure 3 (left panel), unlike controls, SM23-treated biofilms showed large zones with few or no bacteria and scant extracellular matrix onto the well surface. Moreover, the residual biofilm showed drastic alterations in its architecture (Figure 3, left panel). In parallel experiments, a 24 h-old biofilm generated in the presence or absence of SM23 was stained with CFDA and PI, to discriminate live from dead cells, as detailed in section “Materials and Methods.” As shown in Figure 3 (right panel), a significant reduction of viable bacterial cells was observed in biofilms treated with SM23, irrespective of the dose used.

### Inhibition of *Pseudomonas aeruginosa* Quorum Sensing-Related Virulence Factors by SM23

*P. aeruginosa* elastase is a zinc metalloprotease encoded by *lasB* gene, capable of inactivating a wide range of biological tissues and immunological agents (Preston et al., 1997; Li et al., 2019). Thus, we evaluated the elastase activity in supernatants of biofilm producing *P. aeruginosa,* after 6, 12 or 24 h of culture in the presence or absence of scalar doses of SM23. Our results showed a consistent elastase activity by the untreated control cells, already at 6 h persisting up to 24 h of incubation. Such activity was significantly dampened (about 45% of reduction) by SM23 treatment at all the doses tested (Table 1).

**Table 1:** Elastase activity of *P. aeruginosa* in response to SM23 treatment: time- and dose-dependence.

Groups	Elastase activity (OD_495_)		
	6 h	12 h	24 h
Untreated	0.142 ± 0.030	0.114 ± 0.017	0.155 ± 0.013
SM23 (0.780 μM)	0.114 ± 0.011	0.128 ± 0.018	0.092 ± 0.004^*^
SM23 (1.560 μM)	0.103 ± 0.011	0.134 ± 0.018	0.088 ± 0.003^*^
SM23 (3.125 μM)	0.106 ± 0.013	0.109 ± 0.015	0.087 ± 0.006^*^

* *p < 0.05; SM23-treated vs. untreated group, according to Kruskall-Wallis followed by Dunnett’s multiple comparisons test*.

It is widely accepted that pyoverdine, the most potent iron-gathering siderophore in *P. aeruginosa*, is an important virulence factor playing an important role in biofilm formation (Meyer et al., 1996).

HPLC-MS analysis was performed to evaluate the levels of pyoverdines, pyocyanin, 3-oxo-C_12_-HSL and C_4_-HSL under the same experimental conditions used for elastase activity. Pyoverdines, in the form of doubly charged protonated molecular ions, were revealed by the presence of the specific chromophore core (P) fragment ion at *m*/*z* 204.0768 in their MS/MS spectra (Budzikiewicz et al., 2007; Wei and Aristilde, 2015). The identification of the four most intensely detected pyoverdines, achieved using their MS/MS spectra, revealed serine (Ser) as the first amino acid of the peptide sequence and either a succinyl amide (Succa, A1+ fragment ion at *m*/*z* 416.1565 Th) or a succinic acid (Succ, A1+ fragment ion at *m*/*z* 417.1405 Th) moiety as side chain of the chromophore. Two pyoverdines, with their [M + 2H]^2+^ ions being respectively at *m*/*z* 667.8015 and 667.3104 Th, were unambiguously identified, as previously reported for Pyoverdine D (Py D) and E (Py E) (Kilz et al., 1999); differently, other two pyoverdines, portrayed by their respective [M + 2H]^2+^ ions at *m*/*z* 644.7997 and 645.2914 Th, were not yet completely identified and hereinafter referred to as Succ-P-Ser-Y and Succa-P-Ser-Y, with Y being their unidentified peptide sequence side-chain.

Pyocyanin was detected as mono charged protonated ion [M + H]^+^ at *m*/*z* 211.0866 and its identification possibly confirmed by fragments [M + H-CH3]^+^ at *m*/*z* 196.0631, [M + H-CO]^+^ at *m*/*z* 183.0917 and [M + H-CH3-CO]^+^ at *m*/*z* 168.0682 appearing in its MS/MS spectrum (Watson et al., 1986). Both 3-oxo-C_12_-HSL and C_4_-HSL were revealed by the presence of their respective [M + H]^+^ and [M + Na]^+^ molecular ions (at *m*/*z* 172.0968 and 194.0788 for C_4_-HSL and at *m*/*z* 298.2013 and 320.1832 for 3-oxo-C_12_-HSL). Their identification was possibly confirmed by the fragments [M + H-C4H7NO2]^+^ at 71.0491 for C_4_-HSL and at 197.1536 for 3-oxo-C_12_-HSL and [C4H7NO2 + H]^+^ at 102.05496 appearing in their respective MS/MS spectra; these data closely recalled the profile previously established (Churchill et al., 2011).

Compounds elution provided peaks appearing in their specific chromatographic traces, showing the abundance of their respective doubly charged ions over the chromatographic run (mass range chromatograms). Peak areas were used for semi-quantitative evaluation, as previously established (Kapoore and Vaidyanathan, 2016). As depicted in Table 2, we found consistent levels of pyoverdines only after 24 h of *P. aeruginosa* culture. SM23 markedly reduced PyD and PyE production at all the three doses tested, whereas no effect was observed in terms of Py Succ-P-Ser-Y_isom1/2 or Py Succa-P-Ser-Y_isom1/2 levels. Under the same experimental conditions, we analyzed also the release of pyocyanin, another key virulence factor involved in *P. aeruginosa* biofilm formation (Li et al., 2015). Again, we found consistent levels of pyocyanin only after 24 h of *P. aeruginosa* culture, while the treatment with SM23, at 0.780, 1.560, and 3.125 μM, significantly impaired the pyocyanin production.

**Table 2:** Mass spectrometry analysis of *P. aeruginosa* pyoverdine and pyocyanin release in response to SM23 treatment: time- and dose-dependence.

	Py D			Py E		
Groups	6 h	12 h	24 h	6 h	12 h	24 h
Untreated	n.f.	n.f.	21.636.937,83 ± 2.064.621,26	n.f.	n.f.	107.118.272,50 ± 6.827.575,27
SM23 (0.780 μM)	n.f.	n.f.	12.791.348,09 ± 3.054.805,04	n.f.	n.f.	89.272.480,54 ± 9.299.967,42
SM23 (1.560 μM)	n.f.	n.f.	13.394.820,07 ± 3.644.560,57	n.f.	n.f.	78.809.117,60 ± 15.530.420,87
SM23 (3.125 μM)	n.f.	n.f.	11.863.503,70 ± 2.030.689,62	n.f.	n.f.	68.465.442,68 ± 8.162.020,46
	***Py Succ-P-Ser-Y_isom1***			***Py Succ-P-Ser-Y_isom2***		
Groups	6 h	12 h	24 h	6 h	12 h	24 h
Untreated	n.f.	n.f.	12.195.698,41 ± 694.005,68	n.f.	n.f.	13.864.633,08 ± 890.328,25
SM23 (0.780 μM)	n.f.	n.f.	17.853.144,48 ± 1.894.407,84	n.f.	n.f.	20.815.367,39 ± 1.882.888,28
SM23 (1.560 μM)	n.f.	n.f.	17.177.294,38 ± 1.481.504,17	n.f.	n.f.	17.219.927,21 ± 2.725.080,39
SM23 (3.125 μM)	n.f.	n.f.	15.410.576,46 ± 33.533,32	n.f.	n.f.	15.854.231,57 ± 1.705.318,16
	***Py Succa-P-Ser-Y_isom1***			***Py Succa-P-Ser-Y_isom2***		
Groups	6 h	12 h	24 h	6 h	12 h	24 h
Untreated	n.f.	n.f.	1.443.163,09 ± 118.385,60	n.f.	n.f.	2.740.841,46 ± 256.622,87
SM23 (0.780 μM)	n.f.	n.f.	1.745.369,67 ± 327.147,82	n.f.	n.f.	2.791.882,23 ± 154.538,85
SM23 (1.560 μM)	n.f.	n.f.	846.415,5 ± 400.566,41	n.f.	n.f.	2.853.552,30 ± 557.608,16
SM23 (3.125 μM)	n.f.	n.f.	1.133.441,13 ± 30.587,47	n.f.	n.f.	2.205.285,65 ± 499.846,96
	**Pyocyanin**					
Groups	6 h	12 h	24 h			
Untreated	n.f.	n.f.	1.604.817.246,57 ± 56.645.573,69			
SM23 (0.780 μM)	n.f.	n.f.	804.321.499,25 ± 71.053.820,19^*^			
SM23 (1.560 μM)	n.f.	n.f.	945.605.723,51 ± 50.626.110,98^*^			
SM23 (3.125 μM)	n.f.	n.f.	696.891.913,37 ± 34.697.351,25^*^			

*n.f., not found*.

* *p < 0.001 SM23 treated vs untreated group according to one-way ANOVA followed by Dunnett’s multiple comparisons test*.

### Inhibition of *Pseudomonas aeruginosa* Quorum Sensing Molecules by SM23

The results in Table 3 show the levels of both 3-oxo-C_12_-HSL and C_4_-HSL autoinducers in supernatants of *P. aeruginosa* producing biofilm, after 6, 12, and 24 h of SM23 treatment. A consistent presence of 3-oxo-C_12_-HSL was measured already after 12 h that persisted up to 24 h, while C_4_-HSL was detectable only after 24 h of culture. All the SM23 doses significantly reduced 3-oxo-C_12_-HSL after 12 h of treatment; moreover, both the autoinducer molecules resulted significantly inhibited after 24 h of SM23 treatment at the highest dose (3.125 μM).

**Table 3:** Mass spectrometry analysis of *P. aeruginosa* 3-oxo-C_12_-HSL and C_4_-HSL QS molecules release in response to SM23 treatment: time- and dose-dependence.

	3-oxo-C12-HSL			C4-HSL		
Groups	6 h	12 h	24 h	6 h	12 h	24 h
Untreated	n.f.	37.575.520,70 ± 2.315.838,91	201.150.062,20 ± 23.359.699,90	n.f.	n.f.	69.707.031,01 ± 1.143.025,45
SM23 (0.780 μM)	n.f.	23.645.421,84 ± 980.613,63^*^	55.787.081,98 ± 5.098.512,17	n.f.	n.f.	56.319.203,07 ± 2.121.398,89
SM23 (1.560 μM)	n.f.	24.783.514,13 ± 339.890,28^*^	51.160.160,28 ± 1.534.757,47	n.f.	n.f.	57.213.308,48 ± 1.431.287,96
SM23 (3.125 μM)	n.f.	26.816.303,02 ± 1.469.095,35^*^	45.531.599,65 ± 4.653.861,29^*^	n.f.	n.f.	48.215.432,52 ± 3.107.395,93^*^

*n.f., not found*.

12 h: ^*^p ≤ 0.05 SM23 treated vs untreated group according to ANOVA followed by Dunnett’s multiple comparisons test.

24 h: ^*^p ≤ 0.05 SM23 treated vs untreated group according to Kruskall-Wallis followed by Dunnett’s multiple comparisons test.

Finally, we tested the effects of SM23 on *P. aeruginosa* expression of *lasI*/*lasR* genes, known to be crucially involved in QS system, biofilm formation, and virulence (Moghaddam, 2014). Our results showed that, in a 24 h-old biofilm treated with 1.560 μM SM23, both *lasI* and *lasR* gene transcript levels were downregulated by 36 and 25%, respectively (untreated vs. SM23-treated cells: *lasI* returned 1.0 vs. 0.644 fold change; *lasR* returned 1.0 vs. 0.750 fold change).

### SM23 Interaction With *Pseudomonas aeruginosa*

**Figure 4:**
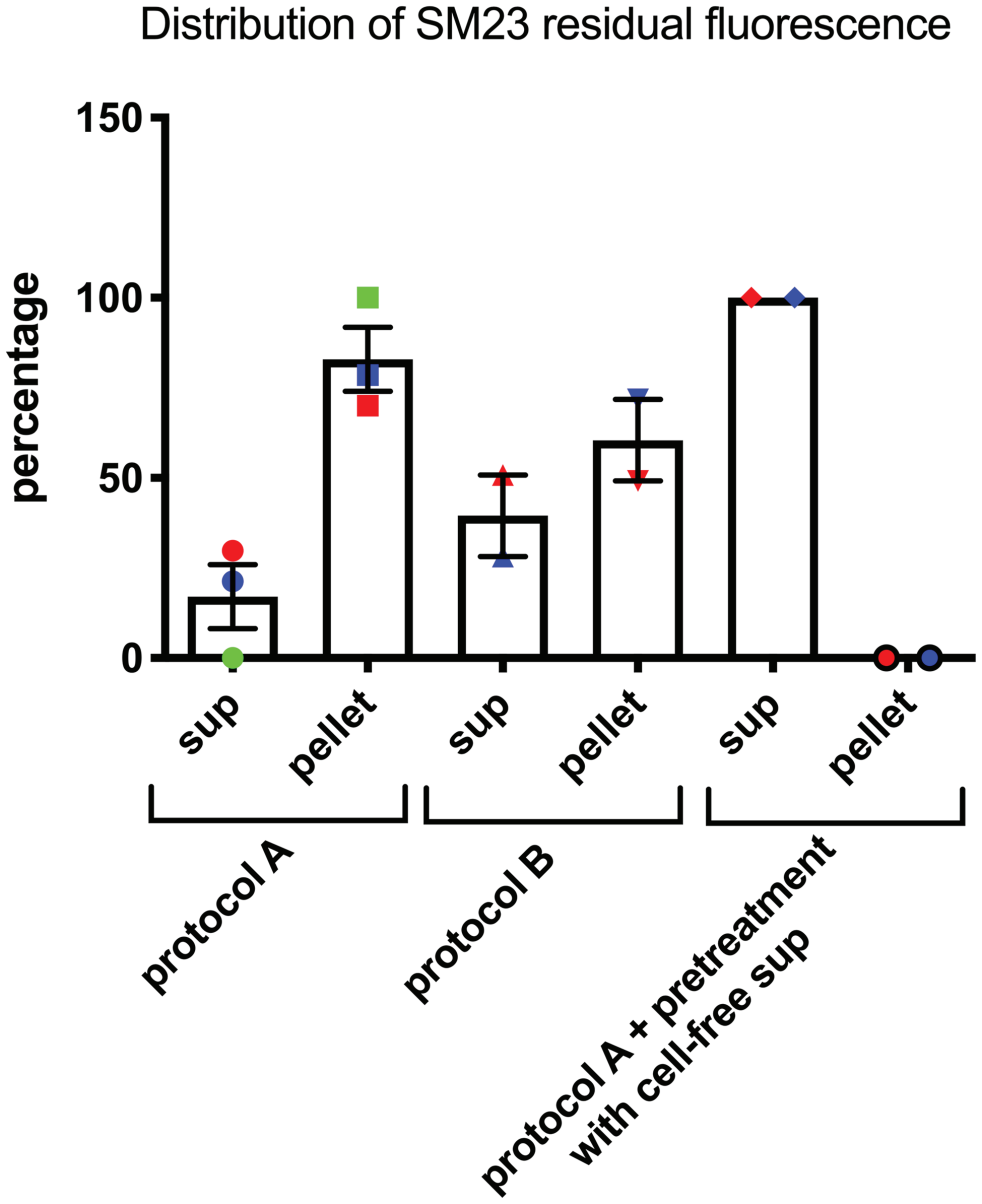
SM23 interaction with *P. aeruginosa* mean percent ± SEM of the fluorescence signal detected both in the cell-free supernatant (sup) and in the pellet. Each percent has been calculated using the data of replicate samples from two-three independent experiments. Each individual symbol identifies a single percent value.

Given the effects of the SM23 on *P. aeruginosa* biofilm formation and *lasI*/*lasR* system, we next investigated its possible mechanism of action. Thus, we developed two different experimental protocols to assess the localization of SM23 on bacterial surface, by exploiting the natural green auto-fluorescence of this compound, as detailed in section “Materials and Methods” and in Figure 1. The results, depicted in Figure 4, showed that, when the SM23 was added at Time 0 (protocol A), most of the fluorescence was associated to the cell pellet, with a minimal residual fluorescence being detectable in the supernatant. In contrast, when the SM23 was added at Time 5 h (protocol B), the fluorescence signal was detected at comparable levels both on the bacterial cell pellet and on the cell-free supernatants.

Finally, a *Pseudomonas*-conditioned medium (from an overnight culture at 37°C) was used to pre-treat the bacterial cells for 30 min; then, the SM23 was added and the plates were further incubated for additional 7 h, as described in protocol A, prior to assessing the levels of free and cell-associated fluorescence. The results, depicted in Figure 4, showed that the fluorescence signal was not cell-associated, but rather only detectable into culture supernatants.

### SM23 Inhibitory Effects on Endotracheal Tubes-Associated Biofilm: Impairment of Biofilm Formation and Pyoverdine Release by *Pseudomonas aeruginosa*

**Figure 5:**
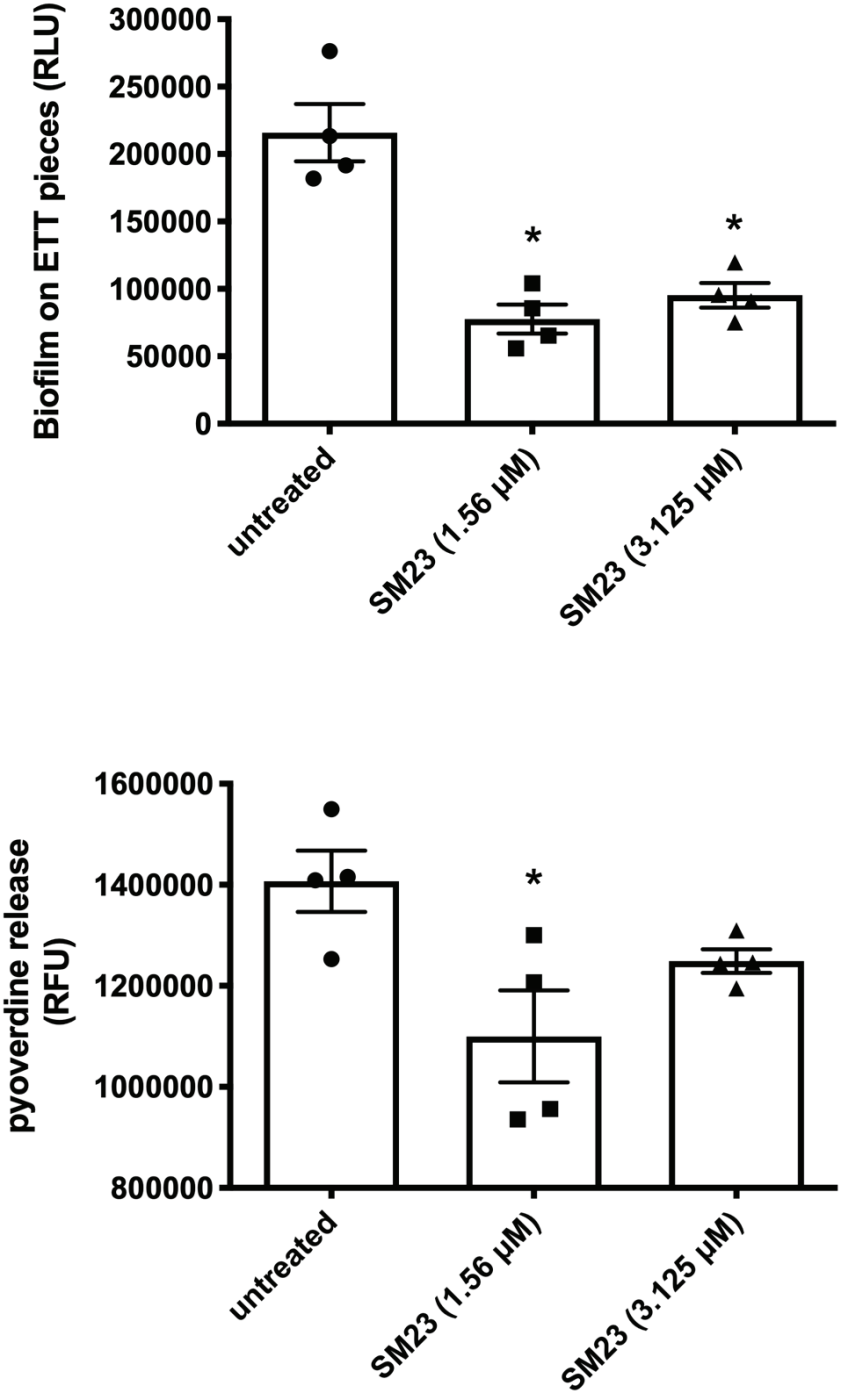
SM23 effects on medical devise-associated biofilm by *P. aeruginosa*: impairment of pyoverdine release and biofilm formation. Biofilm mass (upper panel), expressed as mean RLU ± SEM, and pyoverdine release (lower panel), expressed as mean RFU ± SEM, by a 24 h-old *Pseudomonas* biofilm produced on ETT pieces, in the absence and in the presence of SM23. In the figure, each individual symbol identifies the value corresponding to a single ETT piece. Data represent the mean values from two independent experiments, with duplicate samples. ^*^p < 0.05; SM23-treated samples vs. untreated samples according to ANOVA followed by Dunnett’s multiple comparisons test.

We have recently described a rapid and easy-to-perform *in vitro* model for the real-time monitoring of *P. aeruginosa* biofilm formation on endotracheal tube (ETT) pieces (Pericolini et al., 2018). Accordingly, we evaluated the effect of SM23 on a 24 h-old *Pseudomonas* biofilm produced on ETT pieces. As shown in Figure 5, the treatment with SM23 significantly decreased the amounts of biofilm associated with the ETT, at both the doses used, as indicated by the significant reduction in RLU (Figure 5, upper panel); in addition, the pyoverdine release by such ETT-associated biofilm was also impaired, reaching statistically significant differences at the dose of 1.56 μM (Figure 5, lower panel).

## Discussion

*P. aeruginosa* is a ubiquitous environmental bacterium, responsible for a wide range of severe opportunistic infections, characterized by intense neutrophil activation and significant tissue damage. Clearance is often a serious clinical challenge due to host immunodeficiency and bacterial multidrug resistance, including third-generation cephalosporin (Tenover, 2006; Moghaddam et al., 2012). Moreover, the treatment of chronic *P. aeruginosa* infections is further hampered by adaptive resistance, facilitated also by bacterial ability to form biofilm *in vivo* (Mulcahy et al., 2014). Indeed, bacteria growing as a biofilm have characteristics distinct from their planktonic cell counterpart, including an increased tolerance to antimicrobial agents and host immune response (Costerton, 1999). Furthermore, biofilm is an example of microbial community finely coordinating its behavior through QS. In this study, we provide the first evidence that, SM23, known to be a potent β-lactamase inhibitor, also impairs biofilm formation by *P. aeruginosa* and its production of some QS-dependent virulence factors as well as autoinducer molecules.

The boronic acid derivative SM23 has been described as a strong inhibitor of the *Pseudomonas*-derived cephalosporinase-3 (PDC-3), a class C β-lactamase which represents one of the major antibiotic-resistance determinants in *P. aeruginosa* (*K*_i_ = 4 nM) (Drawz et al., 2011). Such a boronic acid derivative is also able to lower the MIC values of cefotaxime in PDC-3 expressing *E. coli,* to a concentration of 4 μg/ml. Subsequent studies demonstrate that SM23 is highly active against other class C (*K*_i_ of 1 and 20 nM vs. AmpC and ADC-7, respectively) and class A (*β*-lactamases *K*_i_ of 420 and 64 nM vs. CTXM-16 and TEM-1, respectively), qualifying it as one of the best inhibitors of serine β-lactamase (Caselli et al., 2018). Our present results indicate that SM23 significantly inhibits *P. aeruginosa* biofilm formation, as established by measuring total biomass and metabolic activity of a 24 h-old biofilm. In particular, about 50% reduction in biofilm biomass occurs as assessed by CV assay (Figure 2, left panel), while a clear dose-dependent inhibition is detected by bioluminescence analysis; the latter, known to measure metabolic activity, allows to detect a gradual and consistent effect within the range of 0.390–25.0 μM (Figure 2, right panel). The impact of SM23 on biofilm is also evident by exploring its morphological structure by confocal microscopy. Indeed, unlike the control, SM23-treated biofilm is characterized by large zones with few or no bacteria and scant extracellular polymeric matrix attached to the well surface; accordingly, a noticeable reduction of viable bacteria is detected, as measured by the alive/dead cells assay.

Interestingly, the exposure of *P. aeruginosa* to SM23 significantly inhibits the production of some key QS-controlled virulence factors, namely pyoverdine, pyocyanin, and elastase. As a yellow-green fluorescent pigment, pyoverdine is a strong iron scavenger/iron transporter that promotes *Pseudomonas* pathogenicity stimulating its growth (Meyer et al., 1996). To date, about 40 structurally different pyoverdines have been identified, each one characterized by a distinct peptide chain and a specificity, peculiar to *Pseudomonas* species (Meyer, 2000). Pyoverdine production may also be accompanied by the appearance of related compounds, which are considered as biosynthetic precursors or later modifications; such compounds have the same peptide chain of pyoverdine, but a different chromophore group (Budzikiewicz, 2004). Another key pigment strictly regulated by *Pseudomonas* QS system, in particular by *rhlI/rhlR*, is pyocyanin. Here, we show that SM23 strongly inhibits pyocyanin production by *Pseudomonas*, in line with the marked reduction of pyoverdine production also observed after SM23 treatment.

For the determination of both pyoverdines and pyocyanin in culture supernatants, we have employed HPLC-MS analysis, that unambiguously identified (1) four chromophores belonging to the pyoverdine siderophore family and (2) one chromophore for pyocyanin. Interestingly, all of them happen to be markedly affected by SM23 treatment (see Table 2). Similarly, the elastase activity of *P. aeruginosa* is also significantly inhibited by SM23 during biofilm production. In line with these findings, the SM23 is shown to downregulate the expression of the QS-associated autoinducers 3-oxo-C_12_-HSL and C_4_-HSL during biofilm formation by dampened *lasI*/*lasR* gene expression, further suggesting that QS pathways are affected by SM23.

As described previously (Schuster and Greenberg, 2006; Girard and Bloemberg, 2008; Zou and Nair, 2009), the QS system of *P. aeruginosa* is mainly composed of two sets of genes. The first consists of *lasI* and *lasR* genes, encoding for HSL autoinducer synthase and R protein, respectively; the second system, named Rhl, comprises the *rhlI* and *rhl*R genes, which in turn encode for synthase and R protein. The two systems are strictly connected and hierarchically organized, with the *lasI/lasR* regulating the transcription of *rhlI/rhlR* (Schuster and Greenberg, 2006; Zou and Nair, 2009). Our present data, obtained through HPLC-MS analysis, show a significant downregulation of both the autoinducers, 3-oxo-C_12_-HSL and C_4_-HSL, in the supernatants of SM23-treated biofilm (at the dose of 3.125 μM), as compared to the untreated biofilm. These results indicate that SM23 significantly affects the synthesis of both 3-oxo-C_12_-HSL and C_4_-HSL; in line with others (Zou and Nair, 2009), we favor the idea that it may compete with the 3-oxo-C_12_-HSL autoinducer, likely for the same binding site on the LasR receptor. Many efforts will be necessary to deeply understand the molecular mechanisms underlying such a complex phenomenon.

By the experiments aimed at assessing SM23 interaction with bacterial cells, we demonstrate that most of its fluorescence appears to be bacteria-associated when the compound is added to a planktonic *P. aeruginosa* culture. Unexpectedly, such phenomenon is fully prevented once planktonic cells have been pre-incubated with a *Pseudomonas*-conditioned medium, prior to being exposed to SM23; in this case, the compound fluorescence is indeed detectable only in the supernatants. Also, when the compound is added to an early biofilm, approximately half of the SM23-related fluorescence signal remains unbound in the supernatant. Taken together, these data open to the hypothesis that *Pseudomonas* may counteract the activity of SM23 by producing something that prevents its binding to the same bacterial cell. It should be noted that, though strongly associated with the compound, planktonic cells are not affected by SM23 in their viability or rate of growth. Although remaining widely unexplained, these findings may envisage the possibility that, because of its unique mechanism of action, SM23 might be a novel compound likely insensitive to the well-known drug-resistance mechanisms.

Finally, we provide evidence that SM23 is effective also in reducing *Pseudomonas* biofilm biomass produced on a medical device, such as the ETT; importantly, also the release of pyoverdine by such sessile bacterial community is significantly affected by SM23. In our opinion, these data strengthen the interest on SM23, showing that it acts as anti-*P. aeruginosa* compound also under conditions closely mimicking biofilm infection in patients.

Overall, our results demonstrate that the boronic acid derivative SM23, besides being a strong inhibitor of β-lactamase, is able to drastically reduce biofilm formation and QS-related virulence factors in *Pseudomonas*. This *in vitro* evidence opens to future applications of SM23 in the prevention and treatment of biofilm-associated *P. aeruginosa* infections.

## Data Availability Statement

The datasets generated for this study are available on request to the corresponding author.

## Author Contributions

SP, EP, FP, EC, and EB contributed to the conception and design of the study, revised the manuscript critically for important intellectual content, and provided approval for publication of the content. SP, EP, BC, DP, CC, and FF performed experiments and organized the results. EP performed the statistical analysis. SP wrote the first draft of the manuscript. SP, EP, EC, and DP wrote sections of the manuscript. All authors contributed to manuscript revision, read and approved the submitted version.

### Conflict of Interest

The authors declare that the research was conducted in the absence of any commercial or financial relationships that could be construed as a potential conflict of interest.
