# Supplementary material for: The β-Lactamase Inhibitor Boronic Acid Derivative SM23 as a New Anti-Pseudomonas aeruginosa Biofilm
Source: Front Microbiol. 2020 Feb 7;11:35. doi: 10.3389/fmicb.2020.00035 (PMC7018986; doi:10.3389/fmicb.2020.00035)
Supplement: Supplementary file 1 [file Data_Sheet_1.docx]

**Supplementary material**

**SM23 structure**

**Primers list used for real-time PCR analysis**

-16S rRNA FWD: 5’-CGTCCGGAAACGGCCGCT-3’

-16S rRNA REV: 5’-CTCTCAGACCAGTTACGG-3’

- lasI FWD: 5’-AGGCGTGGAGAAGATGATG-3’

-lasI REV: 5’-ATCTGGGTCTTGGCATTGAG-3’

-lasR FWD: 5’-GTGGAAAATTGGAGTGGAGCG-3’

-lasR REV: 5’-GTAGTTGCCGACGATGAAGG-3’
