# Supplementary material for: The β-Lactamase Inhibitor Boronic Acid Derivative SM23 as a New Anti-Pseudomonas aeruginosa Biofilm
Source: Front Microbiol. 2020 Feb 7;11:35. doi: 10.3389/fmicb.2020.00035 (PMC7018986; doi:10.3389/fmicb.2020.00035)
Supplement: Supplementary file 2 [file Data_Sheet_2.docx]

| *P. aeruginosa* clinical isolates | | | |
| --- | --- | --- | --- |
| **Clinical isolates** | **Isolation origin** | **Resistance profile** | **Reference** |
| **Susceptible** |  |  |  |
| ***P. aeruginosa Pa_W10*** | **Skin swab** | **no resistance** | **no publication** |
| **Resistant** |  |  |  |
| ***P. aeruginosa Pa_W6*** | **Diabetic ulcer** | **CAZ-CIP-FEP-FOF-IMP-LVX-MER-PIP-TZP** | **no publication** |
